# Single‐nucleus transcriptomic profiling reveals temporal dynamics of neuroinflammation and myelin repair after intracerebral haemorrhage

**DOI:** 10.1002/ctm2.70486

**Published:** 2025-09-28

**Authors:** Zhan Chen, Qinglin Wang, Rong Xiang, Ruoqi Ding, Jin Tao, Qinfeng Peng, Shaoshuai Wang, Nannan Cheng, Mengke Zhao, Jiaxin Li, Qidi Xue, Chuanyu Liu, Xuemei Chen, Longqi Liu, Junmin Wang, Jian Wang, Mingyue Wang

**Affiliations:** ^1^ College of Life Sciences University of Chinese Academy of Sciences Beijing China; ^2^ BGI Research Hangzhou China; ^3^ Department of Human Anatomy, School of Basic Medical Sciences Zhengzhou University Zhengzhou China; ^4^ BGI Research Shenzhen China; ^5^ Shanxi Medical University‐BGI Collaborative Center for Future Medicine Shanxi Medical University Taiyuan China

**Keywords:** neuroimmune signalling, remyelination, single‐nucleus RNA sequencing, T cell modulation

## Abstract

**Background:**

Intracerebral haemorrhage (ICH) progresses rapidly with complex pathology and limited treatment options, making it a severe subtype of stroke. The extravasation of blood into the brain parenchyma triggers a cascade of inflammatory responses, contributing to secondary injury. Single‐nucleus RNA sequencing (snRNA‐seq) data have enabled more profound insights into the cellular heterogeneity and dynamic interactions within the haemorrhagic brain. Immune cells play a crucial role in shaping neuroinflammation. However, the lack of comprehensive longitudinal studies limits our understanding of the temporal evolution of these inflammatory processes, posing a challenge to the development of targeted therapeutic strategies.

**Methods:**

We used snRNA‐seq in collagenase‐induced ICH mouse models at Days 1, 3, 7, 14 and 28 post‐injury, alongside naive controls, to profile the dynamics of gene expression over time.

**Results:**

We obtained 281 577 high‐quality transcriptional profiles representing 21 distinct cell types. Co‐expression network analysis revealed a prominent ‘inflammation module’ that remained active throughout ICH. Integrative single‐cell transcriptomic and immunofluorescence staining suggested that the various *Mif*‐expressing cells may contribute to local inflammation, potentially engaging macrophages via receptor–ligand pairs such as *Cd44* and *Cd74*. Over time, microglia appeared to serve as key recipients of pro‐inflammatory signals increasingly. During the resolution phase, oligodendrocytes exhibited transcriptional signatures consistent with enhanced maturation and remyelination, which T cell‐mediated interactions may have facilitated.

**Conclusions:**

These findings offer a systems‐level perspective on cell‐type–specific responses and immune‐mediated interactions during ICH progression and resolution.

**Key points:**

- Establish intracerebral haemorrhage (ICH) mouse models at various time points (Days 1, 3, 7, 14, 28) and construct a high‐quality single‐nucleus RNA sequencing (snRNA‐seq) atlas.
- Computational analyses suggest that macrophage recruitment in the early stage of ICH potentially involves migration inhibitory factor (MIF) signalling pathways.
- T cells may interact with myelin‐forming oligodendrocytes during the resolution phase, potentially contributing to remyelination after ICH.

## Graphical abstract

- Establish intracerebral haemorrhage (ICH) mouse models at various time points (Days 1, 3, 7, 14, 28) and construct a high‐quality single‐nucleus RNA sequencing (snRNA‐seq) atlas.
- Activation of astrocytes and microglia drives macrophage recruitment by migration inhibitory factor (MIF) signalling pathways in the early stage.
- T cells promoted remyelination during the resolution phase of neuroinflammation by interacting with myelin‐forming oligodendrocytes.

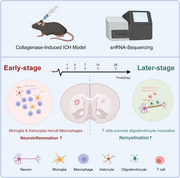

## 1 INTRODUCTION

Intracerebral haemorrhage (ICH) is a severe subtype of stroke, accounting for 27.9% of all strokes worldwide in 2019.^1^ Limited progress has been made in therapeutic strategies for ICH, with only modest improvements in patient outcomes over the past few decades. The pathophysiology of ICH involves direct pressure from the expanding haematoma, as well as secondary physiological and cellular responses triggered by the haematoma and blood‐derived metabolites.^2^ These challenges underscore the pressing need for ongoing research to develop innovative therapeutic strategies for ICH.^3^, ^4^ Blood leakage into the brain during ICH induces a cascade of harmful processes, including brain oedema, thrombin toxicity and neuroinflammation.

Neuroinflammation, mediated by cell types such as microglia, astrocytes, macrophages (MAC) and T cells, is a key driver of secondary brain injury and is associated with poor clinical outcomes.^3^, ^5^ It exacerbates brain damage, hinders tissue repair and contributes to post‐stroke motor and cognitive impairments.^6^ Microglia and MAC, pivotal mediators of inflammation, are traditionally categorised into M1 (pro‐inflammatory) and M2 (anti‐inflammatory) subtypes based on experimental models.^7^ However, accumulating evidence suggests these cells exhibit a functional spectrum rather than discrete states.^8^ Zhang et al.^9^ identified 12 microglial subtypes in peri‐haematomal oedema (PHE) following ICH, revealing diverse phenotypes including DNA repair, degenerative, disease‐associated and transitional inflammatory states. These findings highlight the phenotypic complexity of microglia in the ICH microenvironment.

In addition to neuroinflammation, demyelination is a critical pathological feature of ICH. The breakdown of the myelin sheath disrupts neuronal signal transmission, leading to functional impairments.^10^, ^11^ The process of demyelination can be reversed by oligodendrocyte precursor cells (OPCs), which are vital for restoring myelin integrity and improving recovery. OPCs proliferate and differentiate into mature oligodendrocytes (MOL) to repair damaged myelin.^12^ Emerging evidence also highlights the role of regulatory T cells (Tregs) in promoting remyelination and neural repair by modulating immune responses.^13^

Single‐nucleus RNA sequencing (snRNA‐seq) has enabled a more nuanced understanding of cellular responses to ICH. This technique provides high‐resolution transcriptional profiles of brain cells, facilitating the identification of distinct cellular populations and their molecular pathways.^14^, ^15^ Such insights are crucial for unravelling the complexity of immune cell interactions and their contributions to inflammation.

In this study, we employed snRNA‐seq to investigate the cellular and molecular dynamics of neuroinflammation and remyelination in a collagenase‐induced ICH mouse model. We identified distinct cell clusters and their molecular characteristics, revealing key cellular interactions and regulatory mechanisms involved in inflammation and remyelination. Our findings identify specific therapeutic targets to promote functional recovery after ICH, providing a foundation for developing treatment strategies.

## 2 RESULTS

### 2.1 Transcriptomic landscape of mouse peri‐haematoma tissue

**FIGURE 1:**
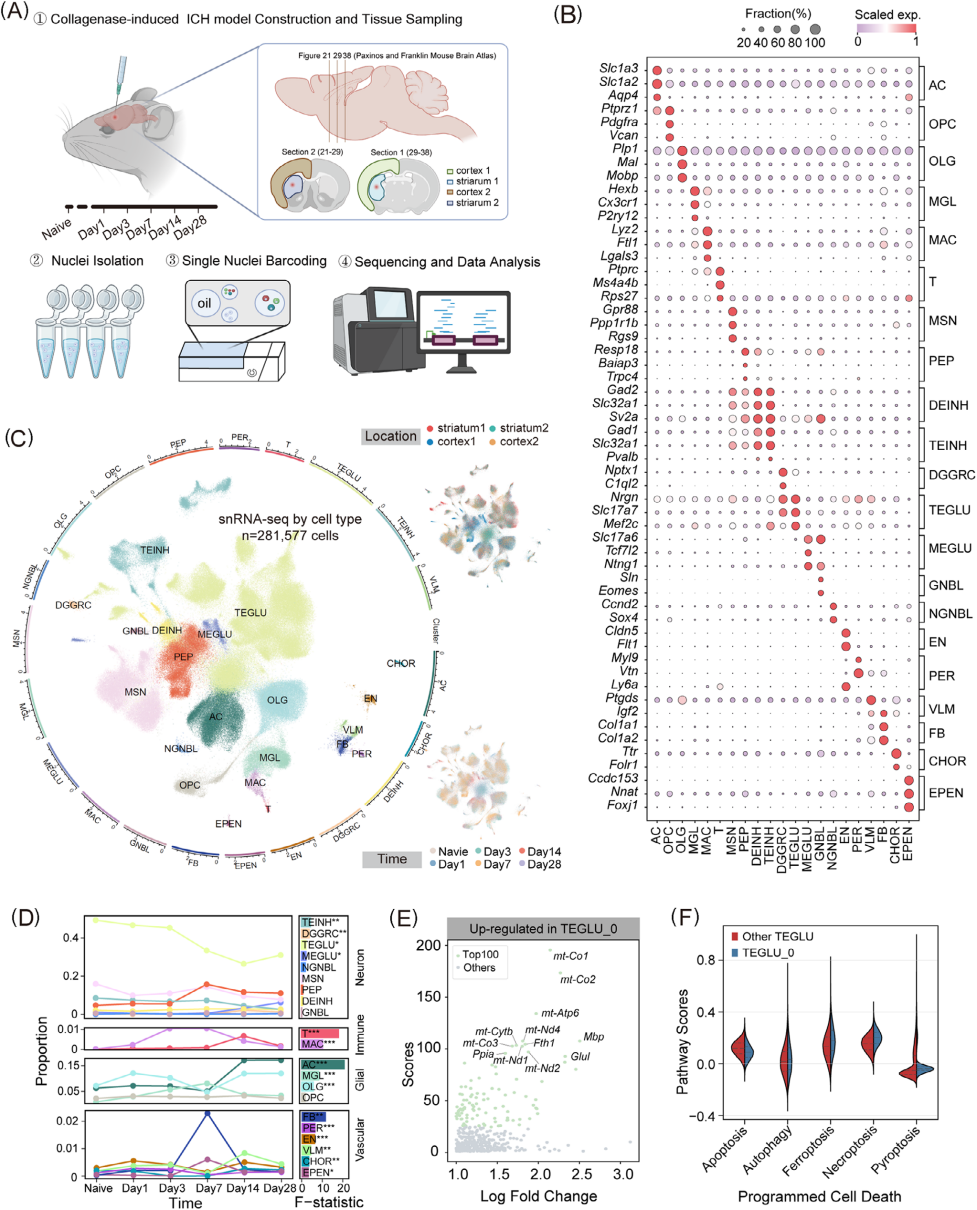
The single‐cell landscape of mouse brain tissue surrounding the lesion in the ICH model and naive controls. (A) Schematic of the workflow for transcriptomic profiling of the ICH model using single‐nucleus RNA sequencing (snRNA‐seq). (B) The dot plot shows the mean expression levels of marker genes for the 21 significant cell populations. The colour represents the average scaled gene expression level (z‐score), and the dot size represents the percentage of cells in which the marker gene was detected for each population. (C) Uniform Manifold Approximation and Projection (UMAP) visualisation of clustering in the snRNA‐seq transcriptomic data from the ICH mouse brain lesion tissue. The outer circles represent the log10‐transformed cell numbers for each cell type. A total of 21 cell populations are identified: DGGRC (dentate gyrus granule neurons), MEGLU (di‐ and mesencephalon excitatory neurons), DEINH (di‐ and mesencephalon inhibitory neurons), GNBL (glutamatergic neuroblasts), NGNBL (non‐glutamatergic neuroblasts), PEP (peptidergic neurons), TEINH (telencephalon inhibitory interneurons), TEGLU (telencephalic glutamatergic neurons), MSN (telencephalon projecting inhibitory neurons), MAC (macrophages), T (T cells), AC (astrocytes), MGL (microglia), OPC (oligodendrocyte precursor cells), OLG (oligodendrocytes), EPEN (ependymal cells), CHOR (choroid plexus epithelial cells), EN (endothelial cells), PER (pericytes), FB (fibroblasts) and VLM (vascular leptomeningeal cells). (D) Line plots (left) show the changes in relative proportions of primary cell types across six time points (naive, Day 1, Day 3, Day 7, Day 14 and Day 28). Bar plots (right) show *F*‐statistics using analysis of variance (ANOVA), coloured by cell types. They are categorised into four major groups: neurons, immune cells, glial cells and vascular cells. (E) The scatter plot illustrates the DEGs of TEGLU_0, with the top 100 genes highlighted in light green. (F) The violin plot shows the programmed cell death pathway scores in TEGLU subtypes.

To examine the temporal dynamics of gene expression following ICH, we utilised the collagenase‐induced ICH model. This well‐established methodology triggers ICH by breaking down the vascular basement membrane.^16^ We monitored gene expression at critical time points after ICH (1, 3, 7, 14 and 28 days). We digested brain sections around the lesion for snRNA‐seq analysis (Figure 1A and Table S1). Cells were filtered based on the mitochondrial gene ratio, gene count and doublet score to remove low‐quality cells (Figure S1A). A total of 281, 577 cells were identified, with an average of 2010 genes detected per cell.

Initially, we used scVI to remove batch effects across libraries from different experimental conditions (see Materials and Methods section). The dimensionality reduction matrix generated by scVI was used for subsequent Uniform Manifold Approximation and Projection (UMAP) and Leiden clustering. We manually identified 67 clusters and annotated them to 21 major cell types using curated marker genes from the literature (Figures 1B, S1B, and Table S2). These cell types included neuronal (9), immune (2), glial (4) and vascular (6) cell clusters (Figures 1C and S2C). Each cell type was present at all time points and locations. However, distinct temporal preferences of cell types were observed (Figure S2A,B). Inhibitory neurons were more abundant in the striatum, while peripheral blood immune cells were comparatively scarce in the naive group. This scarcity was likely due to the intact blood–brain barrier (BBB), which restricted the infiltration of immune cells from the peripheral blood. We observed pronounced diversity within the telencephalic glutamatergic neurons (TEGLU) cluster (Figure S2E), consistent with the cerebral cortex's complex architecture and biological functions.

To investigate shifts in cell types within the lesion after ICH, we analysed their proportions using propeller analysis^17^ (Figures 1D and S2D). The proportion of neuronal cells, particularly TEGLU and TEINH, declined after ICH, which contributes to the death of surrounding neurons. The TEGLU_0 exhibits an increased mitochondrial percentage (Figures 1E and S2F). Previous research indicated that excessive mitochondrial fission exacerbates neuronal pyroptosis and neuroinflammation.^18^, ^19^ We assessed programmed cell death processes using well‐established gene sets.^20^ TEGLU_0 had a higher score than other subtypes in ferroptosis, necroptosis and pyroptosis (Figure 1F). Furthermore, we observed the infiltration of numerous peripheral immune cells into the brain parenchyma following the disruption of BBB, each exhibiting distinct temporal preferences. Specifically, the prevalence of MAC was more pronounced in the early stages, while T cells exhibited a greater presence in the later stages. This observation suggests that peripheral immune cells assume varying biological roles in the progression of ICH.

### 2.2 Cellular contributions to continued activation of inflammatory co‐expression modules

To explain which cell types contributed to the neuroinflammation and examine the molecular dynamics following ICH, we performed a consensus co‐expression network analysis using hdWGCNA. First, we selected the soft‐thresholding power based on the point where the scale‐free topology fit curve (Figure S3A) began to plateau, indicating a good approximation to a scale‐free network while avoiding excessively high powers that would reduce network connectivity. We determined the following optimal thresholds: Naive (5), Day 1 (6), Day 3 (6), Day 7 (5), Day 14 (5) and Day 28 (7). We then systematically constructed separate networks for the gene expression matrix at each time point, integrated networks and identified gene modules. We identified 10 distinct modules across six networks (Figure S3B). Each module consisted of genes with high co‐expression, likely reflecting coordinated regulatory mechanisms and involvement in shared biological processes.

**FIGURE 2:**
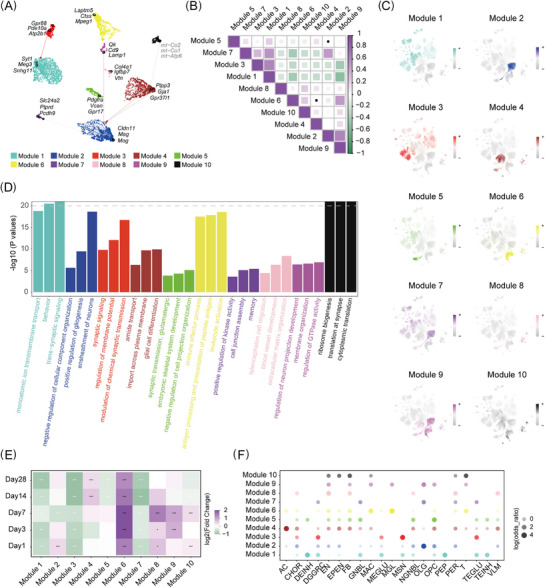
The co‐expression network illustrates changes in gene expression following ICH. (A) UMAP visualisation of the hub gene co‐expression network for the ICH model and Naive group. Nodes, coloured by module assignment, represent genes, with the top three hub genes annotated for each module. Edges, downsampled for clarity, represent co‐expression relationships. (B) Correlation plots show the correlation between each module based on their harmonised module eigengenes (hMEs), with purple indicating positive and green indicating negative correlations. The areas of the squares show the absolute value of the corresponding correlation coefficients. (C) UMAP plot of all cell populations from single‐nucleus RNA sequencing (snRNA‐seq), coloured by module assignment. (D) Bar plot showing the top three gene ontology (GO) enrichment pathways, with the *y*‐axis representing log10 *p* values (values over 20 were truncated for clarity) and bars coloured by module assignment. (E) Heatmap displaying differential module eigengene (DME) results comparing the ICH model to Naive across time points. The Wilcoxon rank‐sum test with Bonferroni correction was applied: not significant (*p* > .05); ∗*p* < .05; ∗∗*p* < .01; ∗∗∗*p *< .001; ∗∗∗∗*p *< .0001. (F) Dot plot showing the enrichment of differentially expressed genes (DEGs) of primary cell types in each module, coloured by module assignment. Dot size represents the logarithm of the odds ratio.

By integrating hdWGCNA and Harmony, we generated harmonised module eigengenes (hMEs), which served as metrics for summarising the gene expression profiles of co‐expression modules. To pinpoint the pivotal genes within each module, we calculated the eigengene‐based connectivity (kEM) of each gene and identified the hub genes, which serve as critical indicators of biological functions (Figure S3C and Table S3). Subsequently, we visualised the co‐expression matrix using hub genes as features, highlighting top three genes (Figure 2A). Module 6 was enriched in pro‐inflammatory genes, while Module 2 exhibited myelination‐related genes (Figure 2D). The edge visualisation indicates that each hub gene exhibits strong co‐expression, underscoring their functional significance.

To investigate potential interactions between modules, we calculated Pearson correlations between them using hMEs. Module 2 and Module 9 notably exhibited strong correlations, with their expressed regions closely aligned with the oligodendrocyte lineage (Figure 2B,C). Interestingly, Module 9 was also expressed in astrocytes and immune cells, suggesting that these cells might support neuronal ensheathment.

We performed differential module eigengene (DME) analysis to identify modules that exhibit time‐specific changes by comparing the ICH groups to naive controls across all time points (Figure 2E). Module 4, primarily enriched in astrocytes (AC) and fibroblasts (FB), exhibited a significant increase after Day 7 (Figures 2F and S3D). Differential expression analysis revealed that several of the top 50 hub genes in Module 4 were up‐regulated in AC at Day 14 compared to the naive group, including genes related to astrocyte maturation process (*Ndrg2, Gpr37l1*) and extracellular matrix remodelling (*Timp3, Sdc4, Clu*; Figure S3E). Moreover, spatial transcriptomic analysis indicated that astrocytes and fibroblasts were located in adjacent regions at the lesion border (Figure S3F).^21^ These findings suggest that astrocytes and fibroblasts cooperatively mediate glial scar formation, thereby contributing to the filling of brain cavities left by the haemorrhage. More importantly, Module 6 showed sustained up‐regulation after ICH compared to the naive group, with elevated expression observed on Days 3 and 7 (Figure 2E). Immune‐related cells, including MAC, microglia (MGL) and T cells, are the primary contributors to this module (Figure 2F). It was enriched in immune effector activity, leukocyte activation and antigen processing and presentation (Figure 2D). The sustained activation of Module 6 suggests that inflammation plays a crucial role after ICH.

### 2.3 Dynamic immune landscape and functional shifts of neuroinflammation cell type during ICH progression

**FIGURE 3:**
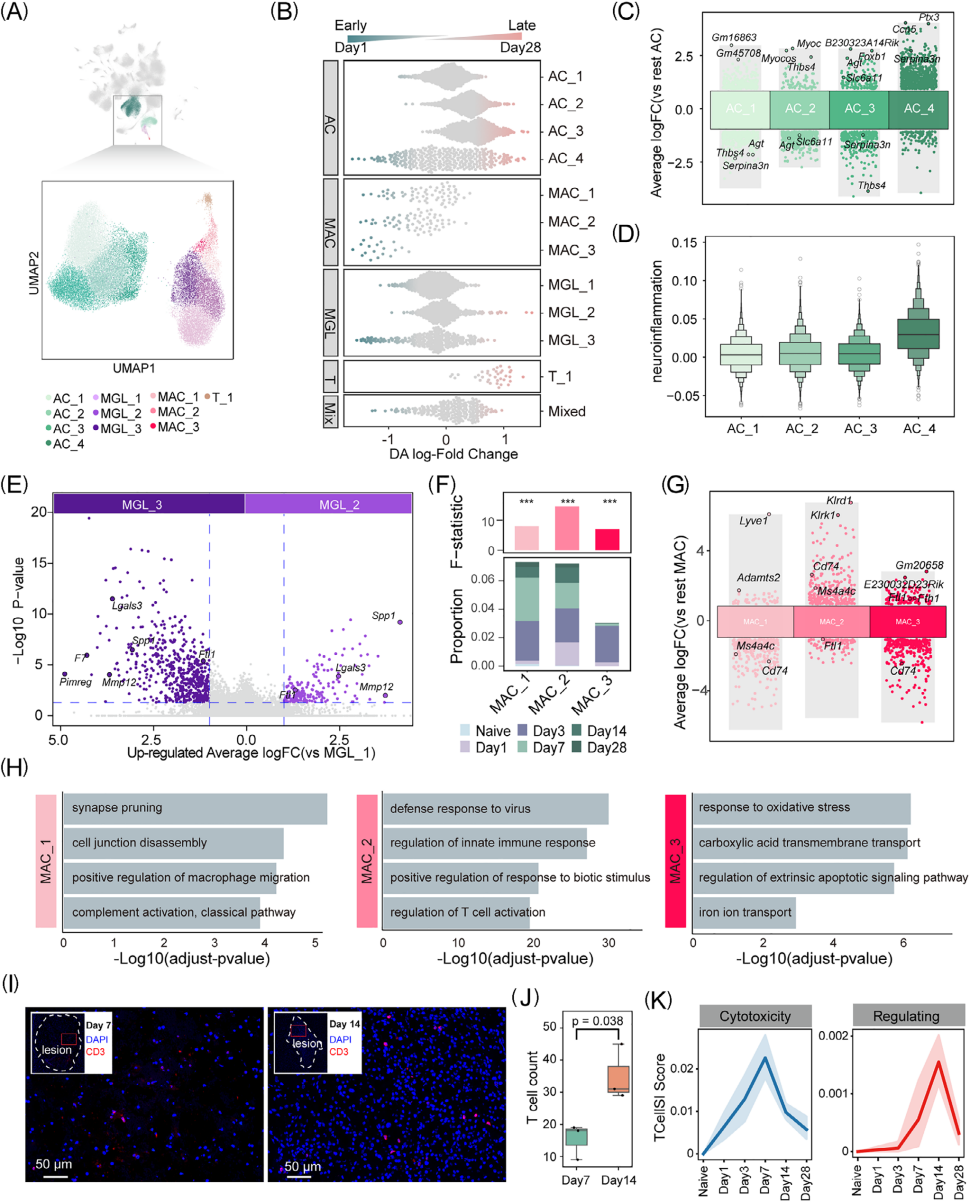
Cellular heterogeneity of neuroinflammation subtypes within the ICH model. (A) UMAP visualisation of subtypes in the snRNA‐seq transcriptomic data from astrocytes (AC), microglia (MGL), macrophages (MAC) and T cells. (B) Beeswarm plot showing differential abundance of neuroinflammation subtypes at each time point after ICH. Significant changes are highlighted in red and blue. (C) Dot plot showing DEGs of astrocytes, coloured by subtype. (D) Box plots showing the neuroinflammation scores of astrocyte subtypes. (E) The volcano plot illustrates the up‐regulated differentially expressed genes of the microglial subtype comparing MGL_1, coloured by subtype. (F) Bar plots (bottom) show the changes in relative proportions of subtypes across six time points (naive, Day 1, Day 3, Day 7, Day 14 and Day 28). Bar plots (up) show *F*‐statistics using analysis of variance (ANOVA), coloured by subtypes. (G) Dot plot showing DEGs of macrophages, coloured by subtype. (H) Bar plot showing gene ontology (GO) enrichment of up‐regulated DEGs in MAC. (I) Representative immunofluorescence images showing CD3⁺ T cells (red) in the lesion core at Day 7 and Day 14 post‐ICH. DAPI (blue) marks nuclei. (J) Box plots showing T cell counts at Day 7 and Day 14 after ICH. *p* value was determined by *t*‐test. (K) Line plots showing inferred T cell functional scores over time after ICH, including cytotoxicity (left) and regulatory (right) programs, as assessed by TCellSI.

Neuroinflammation, a key driver of secondary injury following ICH, remains inadequately understood.^4^, ^22^ Co‐expression network analyses revealed persistent up‐regulation and activation of immune pathways throughout ICH progression. Microglia, astrocytes, MAC and T cells were the principal contributors to the neuroinflammatory response (Figure 2F). The inflammatory scores of these cells were elevated, reaching a peak at Day 7, with MAC and microglia being the primary sources (Figure S4A,B). We conducted a sub‐clustering analysis and identified four astrocyte subtypes, three microglial subtypes and three MAC subtypes (Figures 3A and S4C). We employed milo, a differential abundance (DA) analysis approach based on K‐nearest neighbour (KNN) graphs, to quantify the dynamic changes during ICH progression (Figure 3B). Among immune cells, MAC predominantly appeared at earlier stages. In contrast, T cells were enriched at later stages. AC_4 and MGL_2 exhibited dynamic temporal changes in glial cells, as revealed by their shifting patterns over time (Figure S4D,E). The immune cells infiltrated and induced inflammation after the disruption of the BBB, while the AC and MGL subtypes associated with ICH reflected glial responses to the haemorrhage. These markedly altered subtypes may significantly impact ICH pathology.

To further delineate the molecular signatures of distinct cell subtypes, we analysed differentially expressed genes (DEGs) by pseudo‐bulk and their associated functional pathways (Table S4). AC_2 and AC_3 exhibited gene expression profiles indicative of regionally enriched astrocyte states. Specifically, Thbs4, highly expressed in AC_2, has been associated with white matter astrocytes, while AC_3 expressed Agt and Slc6a11, markers linked to thalamic astrocytes^23^ (Figure 3C). Interestingly, the abundance of AC_4 peaked at Day 7, coinciding with the maximal inflammatory response (Figures 3D and S4F). This cluster exhibited high expression of *Serpina3n*, a gene known to contribute to neuroinflammation by activating the NF‐κB signalling pathway.^24^ It is consistent with previously described reactive astrocyte profiles.^25^ MGL_1, homeostatic microglia, marked by high expression of P2ry12 and Tmem119, which were associated with microglial maintenance (Figure S4C). MGL_3, the earliest microglial subtype to respond to ICH, activated cell cycle pathways to promote cell proliferation (Figures 3E and S4I). MGL_2 was primarily enriched in leukocyte migration and wound healing pathways (Figure S4I).

Peripheral immune cell infiltration after ICH responds to microenvironmental changes within the brain. MAC_3, predominantly on Day 3, cleared iron ions from the haematoma to maintain iron homeostasis (Figure 3F–H). On Day 7, MAC_1 retained a high proportion of cell populations, facilitating the removal of cellular debris by activating complement pathways and immune responses (Figure 3H). MAC_2 showed high expression of Cd74 and was enriched in immune response. Additionally, MAC_2 expressed Ms4a4c, a novel M2 surface marker.^26^ Furthermore, MAC_2 was enriched in positive regulation of T cell activation pathways, further supporting its potential protective function (Figure 3H). The results indicated a functional shift in MAC from iron clearance to debris removal and anti‐inflammation during ICH progression, suggesting that stage‐specific regulation can optimise their therapeutic potential.

Additionally, the number of T cells was insufficient for further sub‐clustering. However, they predominantly appeared during the repair phase, suggesting potential involvement in post‐ICH recovery (Figure S4G). To provide spatial context, we performed immunofluorescence staining for CD3 on brain sections at Days 7 and 14. CD3^+^ T cells were visible in peri‐haematomal regions at both timepoints (Figure 3I,J), qualitatively consistent with their presence during the subacute phase as suggested by single‐cell data. To further explore their potential functions, we applied TCellSI scoring, which revealed a dynamic shift in T cell states: cytotoxicity scores peaked at Day 7, while regulatory scores gradually increased and remained elevated until Day 14, indicating a possible functional transition during recovery (Figure 3K).

### 2.4 Dynamic shift from pro‐inflammatory to anti‐inflammatory signalling by the cell communication of the subtypes

**FIGURE 4:**
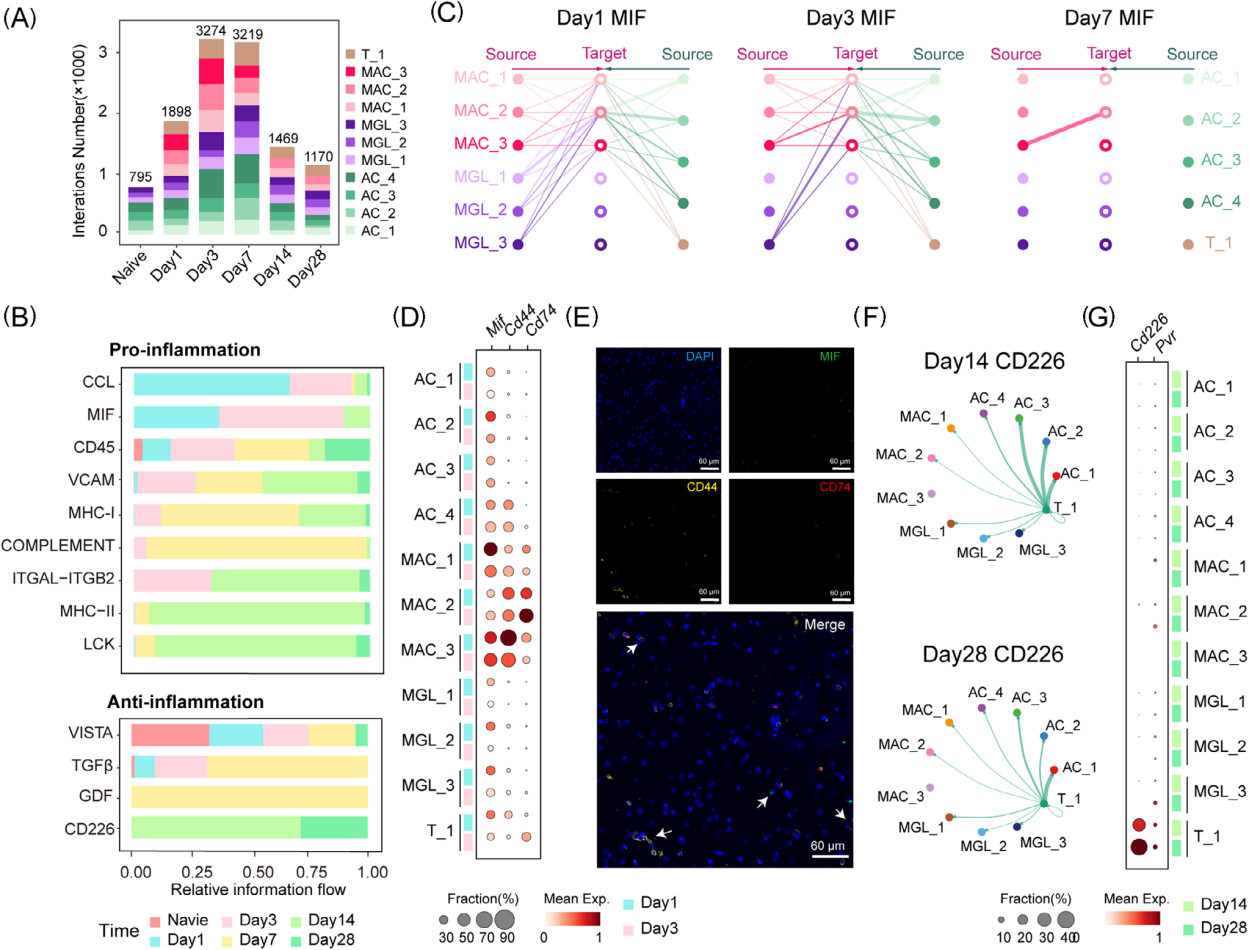
Cell–cell interactions in neuroinflammation subtypes within the ICH model. (A) The number of predicted ligand–receptor (L–R) interactions in neuroinflammation subtypes of ICH, coloured by subtype. (B) The sum of interaction probability differences (relative information flow) for each interaction group in the ICH model, categorised as pro‐inflammatory (top) and anti‐inflammatory (bottom). (C) Hierarchy plot showing the migration inhibitory factor (MIF) signalling network on Day 1 (left), Day 3 (middle) and Day 7 (right). Nodes represent cell types, and edges denote interactions, with width proportional to interaction probability and colour by subtype. (D) The dot plot showing the mean expression levels of L–R of MIF signalling pathway in Days 1 and 3. The colour represents the average scaled gene expression level (z‐score), and the dot size represents the percentage of cells in which the marker gene was detected for each population. (E) Representative immunofluorescence staining shows MIF signalling. MIF is the ligand, and CD44 and CD74 are the receptors. (F) Circle plot showing the MIF signalling network on Day 14 (left) and Day 28 (right). Nodes represent cell types, and edges denote interactions, with width proportional to interaction probability and colour by subtype. (G) The dot plot showing the mean expression levels of ligand–receptor of CD226 signalling pathway in Days 14 and 28. The colour represents the average scaled gene expression level (z‐score), and the dot size represents the percentage of cells in which the marker gene was detected for each population.

To better understand how different cellular subtypes contribute to neuroinflammation, we examined their interactions within the cell communication network, with a focus on pro‐inflammatory and anti‐inflammatory signalling pathways. Notably, the interaction number initially increased until Day 7, following a trend similar to the inflammatory module and scores (Figures 4A and S5A).

The identified pro‐inflammatory pathways exhibited distinct temporal variation patterns (Figure 4B). Specific signalling pathways, particularly those with heightened activity on Day 1, such as the CCL and migration inhibitory factor (MIF) pathways, were of particular interest in elucidating the early inflammatory response. Chemoattractant factors such as Ccl2, Ccl6, Ccl7 and Ccl12 (Figure S5B,C), secreted by diverse cell types within the brain parenchyma, facilitate the recruitment of immune cells to the injury site. Furthermore, MAC expressing *Cd44* and *Cd74* interact with various *Mif*‐expressing cells (Figure 4C,D), suggesting that the MIF‐stimulated CD74–CD44 receptor complex may promote cell survival and proliferation via activation of the ERK pathway.^27^, ^28^ Spatial transcriptomics^21^ revealed a marked accumulation of MAC around the haematoma on the first day after ICH, and *Mif* was broadly expressed by multiple cell types, consistent with our data (Figures 4D and S5D). Immunofluorescence staining further showed spatial proximity between *Mif* signals and *Cd74*/*Cd44* signals (Figure 4E), supporting their potential interaction. These complementary observations indicate that Cd74‐expressing MAC may respond to *Mif* derived from surrounding cells, which may contribute to local inflammatory responses in the acute phase.

During Days 7 and 14 after ICH, we observed enhanced activation of immune cells, as indicated by increased levels of CD45, MHC‐I and complement‐mediated phagocytosis (Figure 4B). CD45 is a marker for activated leukocytes, while MHC‐I presents antigens to *CD8^+^* T cells, promoting immune responses. Complement‐mediated phagocytosis enables MAC to clearing debris and pathogens. In the later stages, pathways, including ITGAL‐ITGB2, MHC class II and LCK, became more prominent. The ITGAL‐ITGB2 pathway was crucial for leukocyte adhesion and migration, facilitating the recruitment of immune cells to the injury site. MHC class II molecules present extracellular antigens to *CD4^+^* T cells, enhancing the adaptive immune response. We compared the signalling intensity of cellular responses to inflammatory pathways at three key time points: the naive group and Days 1 and 7 after ICH (Figure S5E). Our investigation revealed a temporal shift in predominant receptor signalling, with MAC_2 emerging as the primary signalling receptor at the 24‐h post‐ICH time point, followed by a complete transition to MGL‐2 dominance by Day 7 post‐ICH. This shift suggested that managing inflammation in the later stages of ICH necessitated a greater focus on microglial activation.

There was a broader shift from a predominantly pro‐inflammatory response in the acute phase towards a more anti‐inflammatory state in the later stages (Figure 4B). The VISTA pathway, an inhibitory immune checkpoint pathway,^29^, ^30^ was found to be down‐regulated after ICH. The interaction between the microglia‐expressed ligand Vsir and the astrocyte‐secreted protein Igsf11 highlighted the potential role of astrocytes as mediators in modulating inflammatory responses (Figure S5F). We observed an up‐regulation of the TGFβ pathway (*Tgfb2* − (*Tgfbr1* + *Tgfbr2*)) from AC_2, along with the GDF pathway (*Gdf15* − *Tgfbr2*) from T cells on Day 7 (Figures 4B and S5G,H). Both pathways signal through the *Tgfbr2*, which is reported to lead to the suppression of microglial activation.^31^ Additionally, GDF has been shown to regulate triglyceride metabolism and contribute to increased tolerance to inflammatory damage.^32^ Notably, the high expression of *Cd226* on the surface of T cells directly interacts with *Pvr* on inflammatory cells, preventing excessive local inflammation (Figure 4F,G). This interaction is a key regulatory mechanism that maintains local immune homeostasis and protects tissues by balancing positive and negative signals at immune checkpoints.^33^ Anti‐inflammatory pathways were identified within the inflammatory environment, potentially presenting strategies for modulating inflammation following ICH. As we explored this intricate cellular interplay, we gained a clearer understanding of the delicate balance between pro‐inflammatory and anti‐inflammatory signalling.

### 2.5 Oligodendrocyte lineage dynamics and potential T cell–oligodendrocyte interactions after ICH

As neuroinflammation subsides in the later stages of ICH, the resulting shift in the immune microenvironment may create favourable conditions for myelin repair. In the co‐expression network analysis, immune cells contribute significantly to myelin maintenance in Module 9 (Figure 2F).

**FIGURE 5:**
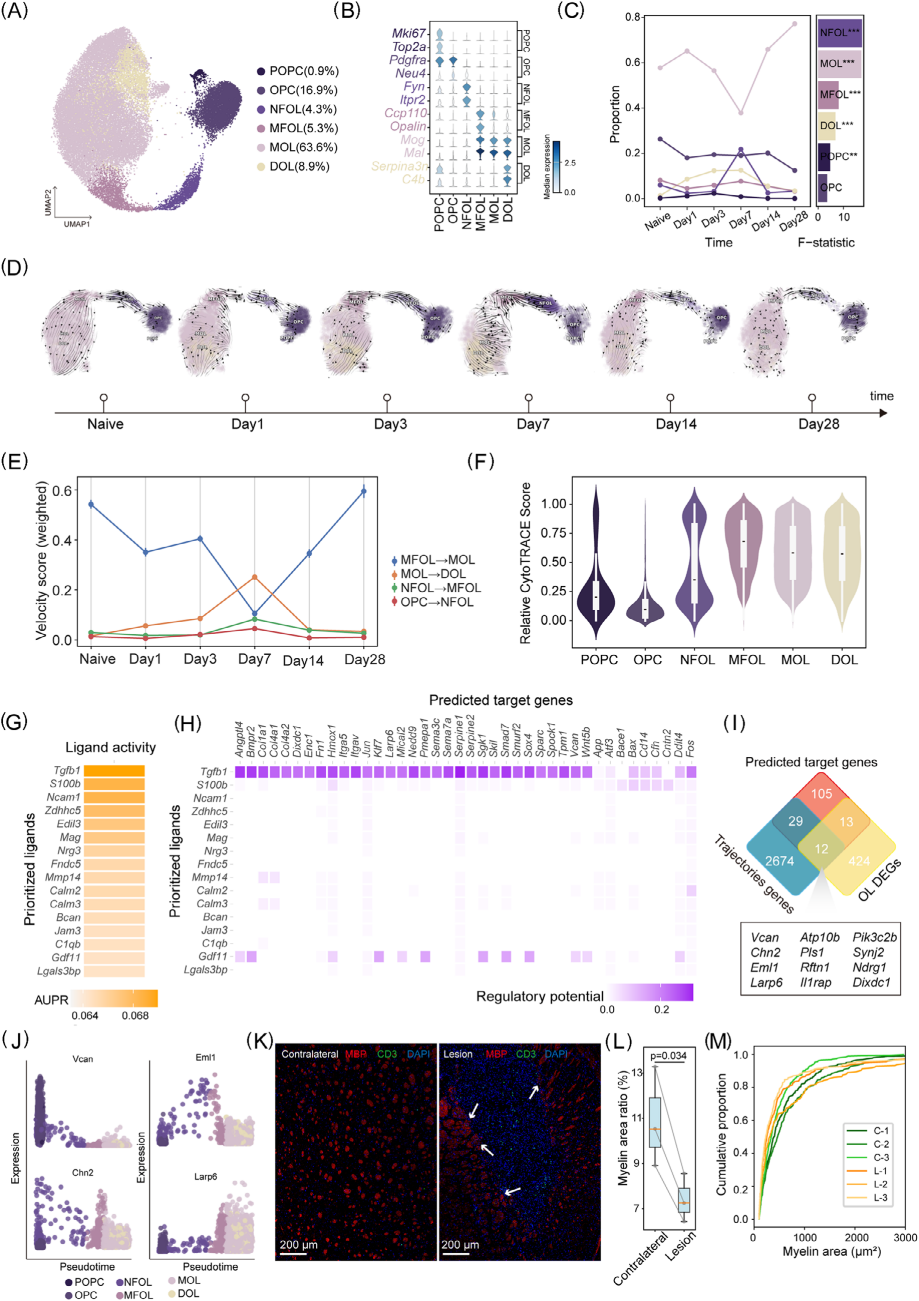
T cells promote the remyelination of oligodendrocyte lineages. (A) UMAP visualisation of oligodendrocyte lineages in the single‐nucleus RNA sequencing (snRNA‐seq) transcriptomic data from OPC and OLG. DOL, disease‐associated oligodendrocytes; MFOL, myelin‐forming oligodendrocytes; MOLL, mature oligodendrocytes; NFOL, newly formed oligodendrocytes; OPCs oligodendrocyte precursor cells; POPCs, proliferative oligodendrocyte precursor cells. (B) Stacked‐violin plot showing the mean expression levels of marker genes for the oligodendrocyte lineages subtypes, coloured by the median expression. (C) Line plots (left) show the changes in relative proportions of subtypes across six time points (naive, Day 1, Day 3, Day 7, Day 14 and Day 28). Bar plots (right) show *F*‐statistics using analysis of variance (ANOVA), coloured by subtypes. (D) Streamline plot showing RNA velocity flow projected in the UMAP space. (E) Time‐resolved quantification of weighted RNA velocity scores across oligodendrocyte lineage transitions after ICH. (F) Violin plot showing the difference potential of oligodendrocyte lineages by the relative CytoTRACE score. The lower score represents the higher potential difference. (G) Heatmap showing ligand activity of T cells, coloured by AUPR (area under the precision–recall curve). (H) Heatmap plot showing the regulatory potential between T and MFOL. (I) Venn plot illustrating the overlap between remyelination trajectories genes and oligodendrocyte lineage cells DEGs in predicting target genes. (J) Scatterplot showing pseudotime dynamics of the expression of *Vcan, Chn2*, *Eml1* and *Larp6* in oligodendrocyte lineage cells. (K) Representative immunofluorescence staining showing CD3^+^ T cells (green) and MBP^+^ myelin (red) in the lesion core (right) versus contralateral hemisphere (left) at Day 14 post‐ICH. (L) Box plots showing quantification of myelin basic protein (MBP) signal in the ipsilateral and contralateral striatum. *p* value was determined by paired *t*‐test. (K) Cumulative frequency curves showing the proportion of myelinated fibres across myelin cross‐sectional areas in the lesion (L‐1, L‐2, L‐3) and contralateral (C‐1, C‐2, C‐3) hemispheres.

To investigate changes in oligodendrocyte lineage cells and the relationship between neuroinflammation. OPCs and OLGs were subdivided into sub‐clusters, identifying six distinct sub‐clusters based on classical markers (Figure 5A,B). We further compared disease‐associated oligodendrocytes (DOL) with MOL (Figure S6C and Table S5). DOL, which increased after ICH, expressed classical injury‐related markers, such as *Serpina3n* and up‐regulated genes, including kallikrein‐related peptidase (*Klk8* and *Klk9*) and fibroblast growth factor receptor (*Fgfr4*). In addition, proliferative oligodendrocyte precursor cells (POPCs) cluster, highly expressed Serpina3n, increased on Days 1 and 3 and had a high cell cycle score (Figures 5C and S6B). The newly formed oligodendrocytes (NFOL) cluster increased on Day 7 (Figures 5C and S6A). The results indicated that OPC accelerated cell cycle under stress and promoted proliferation for myelin repair.

To gain deeper insights into the repair process, we applied Dynamo, a tool rooted in dynamical systems theory and differential geometry, to analyse the spliced mRNA of the oligodendrocyte lineage (Figure S6D). Time‐specific RNA velocity analyses indicate a shift of the oligodendrocyte lineage cells in ICH (Figure 5D). The naive group reflects an active process of oligodendrogenesis. On Day 1, this trajectory is disrupted by ICH, suggesting an early impairment of myelin formation. During the peak of neuroinflammation, particularly at Days 3 and 7, the maturation of NFOL begins to recover. Additionally, we observed two trajectories: one from MOL to DOL, and another from OPC to POPC. These pathways indicate an initial response of oligodendrocyte lineage cells to ICH. Although the differentiation trajectories tend to realign with the naive group during the later stages post‐ICH, they remain more disordered overall, highlighting the long‐term impact of ICH on oligodendrocyte dynamics. To quantify this trajectory, the distribution of RNA velocities was computed at each stage (Figure 5E) and showed a similar trend. We employed CytoTRACE2 to assess the differentiation potential across various sub‐clusters, validating the reliability of the trajectory (Figure 5F).

Previous studies have shown that T cells facilitate myelin repair in multiple sclerosis (MS) by regulating OPC proliferation and differentiation.^34^ However, their specific role in ICH remains largely unexplored. Therefore, we used NichenetR to explore the interactions between T cells and MFOL on Days 7 and 14. Through ligand–receptor analysis, we uncovered that neural cell adhesion molecule (*Ncam1*), which promotes oligodendrocyte survival and process outgrowth following axonal contact,^35^ interacts with *Gfra1*, *Fgfr1* and *Fgfr2* (Figure S6E).

By further investigating the impact of T cell ligands on MFOL target genes, we identified *Tgfb1* (Figure 5G), which has been reported to promote remyelination in the adult CNS in MS.^36^ Our data suggested that *Tgfb1* regulated the expression of multiple target genes in MFOL via receptors like *Tgfbr1*, *Itgb8*, *Itgav* and *App* (Figure S6E). An intersection analysis of the key genes involved in the myelin regeneration trajectory with the target genes identified shared genes. Among these genes, such as, *Chn2*, *Eml1*, *Larp6*, *Ndrg1* and *Vcan*, were highly expressed in oligodendrocyte lineage (Figures 5I and S6F). We further examined representative genes exhibiting dynamic expression patterns along the pseudotime trajectory (Figures 5J and S6G), and found that Vcan and Chn2 were preferentially expressed in early OPC stage. Eml1, late oligodendrocyte differentiation genes,^37^ displayed peak expression in the intermediate differentiation stages. *Larp6*, an oligodendrocyte‐specific RNA‐binding protein (RBP),^38^ exhibited a marked increase in expression specifically at the MFOL stage. Immunofluorescence analysis at Day 14 post‐ICH revealed a dense cellular population within the lesion core, with little myelin present (Figure 5K). Quantification of myelin basic protein (MBP) signal demonstrated a pronounced reduction in total myelin area in the ipsilateral striatum relative to the contralateral side (Figure 5L). Cumulative distribution analysis of individual myelin sheath areas revealed lesion shift towards larger sheath sizes at Day 14 after ICH, which may be attributable to structural loosening secondary to myelin disruption (Figure 5M). At the lesion border, T cells were frequently localised in close apposition to these altered myelin structures (Figure 5K), suggesting a potential cellular interplay between T cells and oligodendrocytes. Although our findings point towards possible T cell–oligodendrocyte interactions during the later stages of ICH, direct evidence for T cell‐mediated myelin repair is still lacking and will require further experimental validation.

## 3 DISCUSSION

We performed snRNA‐seq to profile transcriptomic changes over time in a collagenase‐induced ICH mouse model, focusing on five key time points: 1, 3, 7, 14 and 28 days post‐injury. Compared to previous single‐cell transcriptomic studies, which have primarily focused on the acute phase and concentrated on the haematoma or peri‐haematomal regions, our approach offers a broader temporal perspective. For example, Zhang et al.^9^ conducted scRNA‐seq on human PHE tissue, uncovering considerable immune heterogeneity. In another study, Ye et al.^39^ used a rodent model of white matter haemorrhage to demonstrate shifts in myeloid and OPC populations, highlighting lipid metabolic reprogramming and ferroptosis signalling in association with inflammation. However, one limitation of our study is the use of naive animals as controls rather than sham‐operated animals. While this approach provides a baseline for normal physiological conditions, it does not account for the effects of the surgical procedure itself. Future studies including sham‐operated controls would allow a more precise assessment of ICH‐specific effects.

We identified 67 distinct clusters within the dataset, corresponding to 21 major cell types. Then, we systematically mapped the cell type changes surrounding the lesion site post‐ICH, highlighting their dynamic shifts over time. As a result of the haematoma and secondary injury caused by ICH, the proportion of neurons decreased significantly.^40^ Conversely, the proportions of glial and immune cells exhibited distinct increases at specific time points. Notably, MAC and MGL exhibited prominent increases in the early stages of ICH, whereas AC, OLG and T cells predominantly increased during the mid‐to‐late stages.

Neuroinflammation persists for an extended duration, involving intricate mechanisms even after haematoma clearance, and lacks a clearly defined treatment strategy.^5^, ^41^ Previous studies have indicated that activated glial and immune cells may play vital regulatory roles in neuroinflammation.^10^, ^42^ Through consensus co‐expression network analysis, we observed robust activation of the immune‐inflammatory module throughout the progression of ICH, with the most pronounced changes observed on Day 7. This phenomenon was further validated by evaluating inflammatory pathways. In the initial stages of ICH, the inflammatory response promotes the rapid activation of microglia and the recruitment of MAC to clear damaged cellular debris effectively. However, excessive inflammation leads to prolonged tissue damage, hinders healing and exacerbates secondary injuries.^43^ Despite advancements in our understanding, there are still gaps in our understanding of the precise molecular drivers and the timeline of inflammation's progression.

By sub‐cluster analysis of inflammation‐related cell types, we identified a *Serpina3n^+^* astrocyte subtype that only emerged after ICH, representing injury‐associated astrocytes. Previous studies in epilepsy have shown that *Serpina3n*, released by activated astrocytes, can promote the release of pro‐inflammatory factors via the NF‐κB signalling pathway.^24^ After ICH, MAC and microglia—the brain's intrinsic immune cells—proliferate significantly, contributing substantially to neuroinflammation.^13^, ^44^ Cell communication analyses suggest that MAC recruitment in the early stage may involve MIF‐associated signalling. We revealed the heterogeneity of MAC at various time points after ICH. In the early stage, *Ftl1* ^+^ MAC subtypes primarily clear iron ions and maintain iron homeostasis, which benefits neuron survival.^45^ The *Cd74^+^* and *Ms4a4c^+^* MAC subtypes appeared at later stages, indicating a shift in MAC composition over time. While some progress has been made in understanding the diverse roles of MAC following ICH, the regulatory mechanisms driving shifts in MAC function remain poorly understood. Future research is needed to clarify these mechanisms and to pave the way for novel therapeutic strategies. In addition, microglia respond to ICH by differentiating into two distinct subtypes that may play specialised roles in mediating the inflammatory response. We initially identified a microglial subtype with high proliferative capacity during the early stage. It plays a vital role in compensating for microglial loss resulting from ferroptosis and other cell death mechanisms.^46^ The other microglial subtype exhibits enhanced phagocytic and antigen‐presenting functions, aiding in the clearance of haematomas and glial scars. After Day 7 of ICH, this subtype becomes the primary target of inflammatory signalling pathways, suggesting that modulating microglial activation is critical for managing inflammation in the later stages of ICH. This shift may be due to a decline in MAC numbers during the repair phase, allowing microglia to assume a more dominant role. Notably, microglia may also contribute to limiting inflammation at this stage. For example, Li et al.^47^ reported that newly repopulated microglia exhibit a reduced inflammatory profile. Similarly, Spittau et al.^48^ demonstrated that TGFβ signalling in microglia plays a crucial anti‐inflammatory role, further supporting the idea that microglia become key regulators of inflammation in the subacute to chronic phases after ICH. Regulating immune cell activation and their transitions between functional states presents promising therapeutic targets for managing neuroinflammation after ICH.^7^ Elucidating the mechanisms behind these transitions can inform strategies to modulate immune responses, potentially mitigating inflammation and facilitating tissue repair.

Focal inflammatory lesions can lead to substantial white matter damage, often resulting in demyelination, which disrupts the function of neural cells.^49^ Recent studies suggest that myelin repair is associated with newly differentiated and MOL.^50^ We identified the complete oligodendrocyte lineage and observed the responses of OPC and OLG to the brain injury microenvironment after ICH. DOL, enriched in leukocyte migration and immune responses, exhibited high expression of *Serpina3n*, a signal associated with demyelination.^10^, ^51^ *Serpina3n* was also highly expressed in POPC, suggesting that the altered microenvironment may influence the OPC cell cycle, stimulating their proliferation. Our RNA velocity analyses indicate that ICH leads to early disruptions in oligodendrogenesis, followed by enhanced differentiation at later stages, which may reflect an intrinsic regenerative mechanism contributing to myelin repair during inflammation resolution.

The role of Tregs in promoting myelin formation is an emerging area of academic interest.^52^, ^53^, ^54^ We evaluated T cells in our dataset and found they shifted from cytotoxic to regulatory functions in the later stages of ICH. Interactions between T cells and MFOL identified pathways such as *NACM1* and *FGFR2*, which support cell survival and myelin formation. *TGF‐β1* was identified as an essential ligand for myelin maintenance. Its potential target gene is *Larp6*, an OLG‐specific RBP. However, the scarcity of T cells makes it impossible to distinguish the effects of different subtypes more precisely. Additionally, while Larp6 suppressed sphingomyelin synthesis in colorectal cancer,^55^ further pathway experiments are essential to confirm its role in myelin regeneration in future.

Overall, our study advances the understanding of the single‐cell transcriptomic landscape of ICH and provides insights into the underlying molecular mechanisms following ICH.

## 4 MATERIALS AND METHODS

### 4.1 ICH mouse model

Based on our established protocol for ICH modelling,^56^ in this study, male C57BL/6 mice were anaesthetised with isoflurane in a mixture of 70% N_2_O and 30% O_2_ (4% induction and 2% maintenance). The mice were placed in a stereotaxic frame, and a 1–1.5 cm incision was made along the sagittal suture. After filling the incision area with paraffin wax, a glass micropipette connected to a micro‐infusion pump was used to inject collagenase VII‐S (.075 U in.5 µL sterile saline; Sigma) into the left striatum at coordinates.8 mm anterior to bregma, 2.0 mm lateral to the midline and 3.5 mm deep from the skull surface, using a dual‐arm brain stereo locator (Stoelting, 51503D; Stoelting Co.). The collagenase was infused at a constant rate of.1 µL/min using the micro‐infusion pump (Stoelting). The mice's body temperature was maintained at 37.0 ± .5°C during the procedure using a body temperature maintenance device (RWD). The naive group consisted of untreated male C57BL/6 mice, and any mice that died within 24 h post‐surgery were excluded from the analysis.

The ethical approvals for this study were obtained from the Life Science Ethics Review Committee of Zhengzhou University (Approval No. ZZUIRB 2022–148) and the Institutional Review Board of BGI ETHICAL CLEARANCE (Approval No. BGI‐IRBE23025).

### 4.2 Tissue collection and single‐nucleus isolation

Brain tissues were collected from ICH model mice at Days 1, 3, 7, 14 and 28, with one mouse assigned to each time point. Brain sections were obtained from regions surrounding the haematoma. Specifically, brains from the ICH model mice were placed in a brain matrix, and the cortex and striatum were dissected at predefined coordinates—Section 1 (Figures 29–38 of the Paxinos and Franklin Mouse Brain Atlas) and Section 2 (Figures 21–29 of the Paxinos and Franklin Mouse Brain Atlas). This approach ensured anatomical consistency and reproducibility in sampling regions adjacent to the haematoma. The collected tissues were immediately snap‐frozen for subsequent snRNA‐seq. Single‐nucleus isolation was performed following a previously described protocol with slight modifications.^57^ Briefly, the thawed tissues were minced and mixed with a lysis buffer (10 mM Tris–HCl, pH 7.4; 10 mM NaCl; 3 mM MgCl_2_;.1% IGEPAL CA‐630; 1% BSA; 1× protease inhibitor; and 1 U/µL RNase inhibitor). The mixture was transferred to a 1‐mL Dounce homogeniser and homogenised with 25–50 strokes using a loose pestle (pestle A), followed by 25 strokes with a tight pestle (pestle B). The homogenate was filtered through a 40‐µm strainer and centrifuged at 500 × *g* for 5 min at 4°C to pellet the nuclei. The nuclei were stained with 4′,6‐diamidino‐2‐phenylindole (DAPI) and counted using a Countstar automated cell counter to determine the concentration of the nuclear suspension. Based on the counting results, an appropriate volume was taken to adjust the final concentration to 1000 nuclei/µL in a cell resuspension buffer for library preparation.

Following the manufacturer's protocol, the DNBelab C Series Single‐Cell Library Prep Set (MGI Tech Co.) was used for preparing snRNA‐seq libraries. In brief, single‐nucleus suspensions were used for droplet generation, emulsion breakage, bead collection, reverse transcription and cDNA amplification to create barcoded libraries. Indexed libraries were then constructed according to the manufacturer's protocol. Concentrations were measured using a Qubit ssDNA Assay Kit (Thermo Fisher Scientific, Q10212). The libraries were quantified using a Qubit single‐strand DNA Assay Kit (Invitrogen) and sequenced with paired‐end reads on the DNBSEQ platform at China National GeneBank (Shenzhen, China), employing the following sequencing strategy: 41‐bp read length for read 1 and 100‐bp read length for read 2.

### 4.3 Quantification and statistical analysis

#### 4.3.1 Raw data processing

Raw sequencing reads from DNBSEQ‐T10 were filtered and demultiplexed using PISA (Version 1.1). Before alignment, Drop‐seq (Version 1.13) was used to trim poly(A) stretches, append cell and UMI barcodes to the reads and filter out reads with low‐quality barcode bases. Reads were aligned to the GRCm38 genome using STAR (Version 2.7.4a) and sorted with Sambamba (Version 0.7.0). For snRNA‐seq data, both exon and intron reads were combined to count each gene's transcripts. Finally, a nucleus versus gene UMI count matrix was generated using PISA.

#### 4.3.2 Doublet removal

Doublet removal for each library was performed using Scrublet (Version 0.2.3).^58^ Scrublet detects doublets by simulating artificial doublets within the dataset and constructing a nearest‐neighbour classifier without requiring prior knowledge or pre‐clustering. Doublet removal was conducted using Scrublet's default parameters, excluding the top 5% of cells that showed the highest similarity to simulated pseudo‐doublets.

#### 4.3.3 Lower‐quality cell removal

We applied the median absolute deviation (MAD) method for each library to dynamically identify and remove outlier cells from the dataset. Specifically, MAD was calculated based on UMI counts, gene numbers, mitochondrial percentage and ribosomal percentage, using a threshold of five times the MAD to determine which cells to retain. Additionally, we imposed basic quality control criteria, requiring UMI counts above 1000, gene numbers exceeding 500 and mitochondrial percentages below 10% to ensure data quality.

#### 4.3.4 Ambient RNA removal

Ambient RNA noise was reduced using the ‘DecontX’ function of celda (Version 1.18.1) with default settings. This function was applied to estimate and remove contamination from single‐cell genomic data.

### 4.4 Integration, clustering and cell‐type annotation

After obtaining the filtered gene UMI count matrices, we employed a semi‐supervised pipeline for preliminary annotation. We utilised Scanpy (Version 1.10.2) to merge all sample data and subset the matrix of highly variable genes. Next, we employed scVI, a deep‐learning approach, to remove batch effects from the data. Based on the batch‐corrected dimensionality reduction matrix, we performed clustering using the Leiden algorithm, setting the resolution parameter to 5. Shi et al.’s datasets were used to construct our model and annotate our datasets accordingly,^59^ followed by manual curation of the annotations. All literature cited in Table S2 has been independently verified and can be traced via the provided PMID.

### 4.5 Cell cluster proportion analysis

Cell cluster proportions among different groups were compared using one‐way analysis of variance (ANOVA) followed by Dunnett's multiple comparison test. All statistical analyses were performed in R using the speckle package (version 1.0.0).

### 4.6 Consensus co‐expression network analysis

To investigate global transcriptional changes at the gene expression level, we applied the hdWGCNA (Version 0.3.03)^60^ to perform Consensus Co‐expression Network Analysis, following tutorials from https://smorabit.github.io/hdWGCNA/index.html. Additionally, DME analysis was performed to identify modules that are up‐regulated or down‐regulated after ICH.

### 4.7 Neighbourhood‐based differential abundance analysis

We used the milopy (Version 0.1.0) framework to detect dynamic changes in fine‐grained cell populations over time. Neighbourhoods were constructed from the KNN graph and DA testing was performed using a linear model. To capture temporal progression, time points (D1, D3, D7, D14, D28) were ordinally encoded as integers and used as a continuous variable in the design formula.

### 4.8 Subtype analysis, DGE analysis and Gene Ontology (GO) analysis

To further investigate the biological functions of the cell types of interest, we conducted a subgroup analysis using Scanpy for transcriptomic data for the AC, MGL, MAC, T, OLG and OPC, respectively. Based on the number of cells in each group and their biological functions, we performed multiple rounds of testing to determine an appropriate resolution. Subsequently, differential expression analysis was performed using the Libra R package (version 1.7), which implements a pseudo‐bulk approach to control for biological replicates in single‐cell RNA‐seq data. Raw gene counts were first aggregated across cells from the same cluster within each sample to construct a pseudo‐bulk expression matrix. Comparisons were then made between defined cell subtypes and their respective reference populations using the run_de() function in Libra, which internally utilises DESeq2 for modelling and statistical testing. Genes with a *p* value <.05 and absolute log_2_ fold change >1 were considered significantly differentially expressed. Additionally, we conducted enrichment analysis for up‐regulated genes across multiple groups using the ‘compareCluster’ function from the clusterProfiler (Version 4.12.6).^61^

### 4.9 Cell–cell communication

We utilised the CellChat package (Version 1.5.0)^62^ to analyse communication networks within the neuroinflammation subtypes. We employed the entire cellChatDB.mouse as the receptor–ligand database to identify potential interactions. We used the key function ‘computeCommunProb’ with the ‘truncatedMean’ method to calculate the average gene expression for each cell subtype during the critical interaction analysis. We followed tutorials from https://github.com/sqjin/CellChat and built the cell–cell communication network for each time point, identifying the pathways using the ‘netP’ data slot. Finally, we focused on the signalling pathways related to neuroinflammation.

### 4.10 Multi‐scoring analysis

To describe the continuous spectral variation between different cell types, we conducted various scoring analyses on our essential data. First, we analysed the inflammatory response levels of immune cells using the ‘score_genes’ function in Scanpy, based on the genes associated with the inflammatory response (GO:0006954), with the Naive group serving as a calibration baseline.

Additionally, we assessed the differentiation potential of oligodendrocyte lineages and scored the cell cycle phases. For differentiation potential, we utilised the CytoTRACE2 (Version 1.0.0)^63^ with default parameters to process the raw count matrix, yielding differentiation potential scores for each cell. The cell cycle scoring was performed using the ‘score_genes_cell_cycle’ function in Scanpy to assess S phase and G2M phase scores.

Finally, we evaluated the functional scoring of T cells by employing the TCellSI (Version 0.1.0).^64^ This scoring was based on the standardised expression matrix using the ‘TCSS_scRNAseqCalculate’ function. This process utilised HomoloGene (Version 1.4.68.19.3.27) to convert homologous genes between humans and mice.

### 4.11 RNA velocity and vector field analyses

We reconstructed the expression dynamics vector fields using dynamo (Version 1.3.2),^65^ following guidelines provided by the official tutorials at https://dynamo‐release.readthedocs.io/en/latest/. Briefly, we used the spliced and unspliced counts matrix from the snRNA‐seq data of the oligodendrocyte lineage. RNA velocity, acceleration and curvature were then calculated for demyelination and remyelination. The effects of RNA velocity on remyelination following the perturbation of specific genes were simulated by setting the expression levels of these genes to 25.

### 4.12 Transition velocity quantification across the oligodendrocyte lineage

To quantify transition velocities along the oligodendrocyte lineage, we used the Pearson transition matrix. For each cell at a given state, we computed the summed transition weights towards cells in the subsequent state (e.g., from OPC to NFOL), using a minimum weight threshold to filter spurious connections. This score represents the potential for transition towards the target state. We repeated this procedure across defined lineage stages (OPC → NFOL → MFOL → Moll → DOL) and across developmental time points. Transition velocity scores were aggregated and plotted by time point and lineage transition.

### 4.13 NicheNet analysis

To explore T cells driving myelination, we utilised Nichenetr (Version 1.0.0)^66^ to identify potential ligands mediating interactions between T cells and both NFOL and MFOL. First, we designated NFOL and MFOL as the receivers and identified genes expressed in at least 5% of the cells, focusing on DEGs compared both Day 7 and Day 14 with the Naive stage. We then predicted active ligands based on background genes, inferred corresponding receptors and target genes and identified the top‐ranked ligands. Finally, we pinpointed key target genes for subsequent perturbation analysis.

### 4.14 Immunofluorescence

Multiplex immunofluorescence staining was performed on paraffin‐embedded brain sections using a tyramide signal amplification (TSA)‐based kit (#AFIHC027, AiFang Biological Inc.). Following deparaffinisation and antigen retrieval in citrate buffer (pH 6.0), sections were incubated with primary antibodies overnight at 4°C. After washing, HRP‐conjugated secondary antibodies were applied, followed by TSA fluorophore amplification. Between each staining round, antibody stripping was performed using elution buffer (#G1266, AiFang), allowing sequential detection of multiple targets. Nuclei were counterstained with DAPI, and slides were mounted with antifade mounting medium.

The following panels were used:

1. MIF signalling pathway (Figure S5D): A five‐colour panel including CD44 (1:1500, # AF14754, AiFang Biological Inc.), CD74(1:1500, #AF06806, AiFang Biological Inc.), MIF (1:1500, #AF05273, AiFang Biological Inc.), TMEM119 (1:1500, ab209085, abcam) and F4/80 (1:1500, #SAD002, AiFang Biological Inc.).
2. MIF signalling pathway (Figure 4E): A three‐colour panel targeting MIF (1:1000, BS‐1044R, Bioss), CD74 (1:8000, GB115427, Servicebio) and CD44 (1:2000, GB112054, Servicebio).
3. T cell infiltration (Figure 3I): CD3 (1:2000, #GB12014, Servicebio).
4. T cell–OLG crosstalk (Figure 5K): A dual‐labelling panel targeting CD3 (1:2000, #GB12014, Servicebio) and MBP (1:5000, #GB12226, Servicebio).

Images were acquired using a multispectral fluorescence scanner equipped with appropriate filters.

### 4.15 Immunofluorescence analysis

The red channel corresponding to myelin staining was extracted and converted into a binary mask by applying an appropriate threshold. The resulting mask was refined by morphological operations (opening, closing and hole filling), and regions of interest were further corrected manually in the ROI Manager. Quantification was performed with the ‘Analyse Particles’ function, with a minimum size threshold of 100 µm^2^ to exclude small noise signals. The total myelin‐positive area and its fraction relative to the image field were recorded. T cell numbers were counted manually in the same software.

## AUTHOR CONTRIBUTIONS

Jian Wang, Mingyue Wang and Junmin Wang conceived the study. Mingyue Wang, Qinfeng Peng, Shaoshuai Wang, Nannan Cheng, Mengke Zhao, Jiaxin Li and Qinglin Wang performed experiments. Zhan Chen, Qinglin Wang, Rong Xiang and Jin Tao analysed and interpreted the data. Zhan Chen, Junmin Wang, Jian Wang and Mingyue Wang drafted and revised the manuscript. Ruoqi Ding performed the verification experiments. Longqi Liu, Chuanyu Liu and Xuemei Chen provided advice. All the authors reviewed and approved the manuscript.

## CONFLICT OF INTEREST STATEMENT

The authors declare no conflicts of interest.

## ETHICS STATEMENT

All animal experiments were approved by the Institutional Animal Care and Use Committees of Zhengzhou University (approval No. ZZUIRB 2022‐148) and BGI Research Institute (approval No. BGI‐IRB E23025), and were performed in accordance with national guidelines for the care and use of laboratory animals and the ARRIVE guidelines.

## Supporting information



Supporting Information

Supporting Information

Supporting Information

Supporting Information

Supporting Information

Supporting Information

Supporting Information

Supporting Information

Supporting Information

Supporting Information

Supporting Information

## Data Availability

All data generated in this study are freely available in the China National GeneBank DataBase (CNSA) at https://db.cngb.org/cnsa/download/ under the accession number CNP0006428. All other data are included in the main manuscript or in the Supporting Information. All codes used in this study are publicly available, with sources cited in the main text and methods. Additional information necessary to reproduce or reanalyse the data reported in this paper is available from the Lead Contact upon reasonable request.
