# Supplementary figures and images for: Single‐nucleus transcriptomic profiling reveals temporal dynamics of neuroinflammation and myelin repair after intracerebral haemorrhage
Source: Clin Transl Med. 2025 Sep 28;15(10):e70486. doi: 10.1002/ctm2.70486 (PMC12477064; doi:10.1002/ctm2.70486)

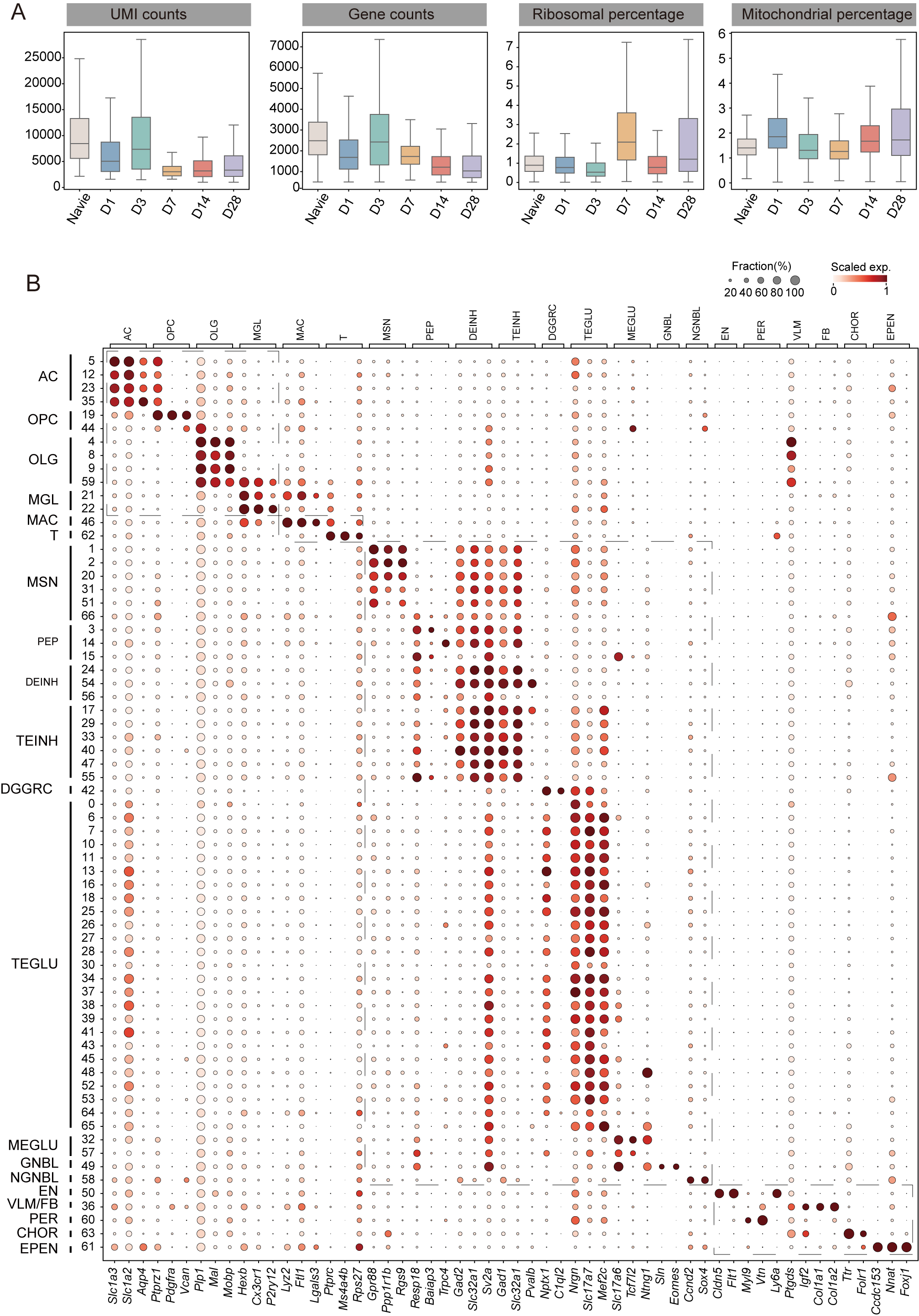

Supplement: Supplementary file 6 — Supporting Information [file CTM2-15-e70486-s009.tif]

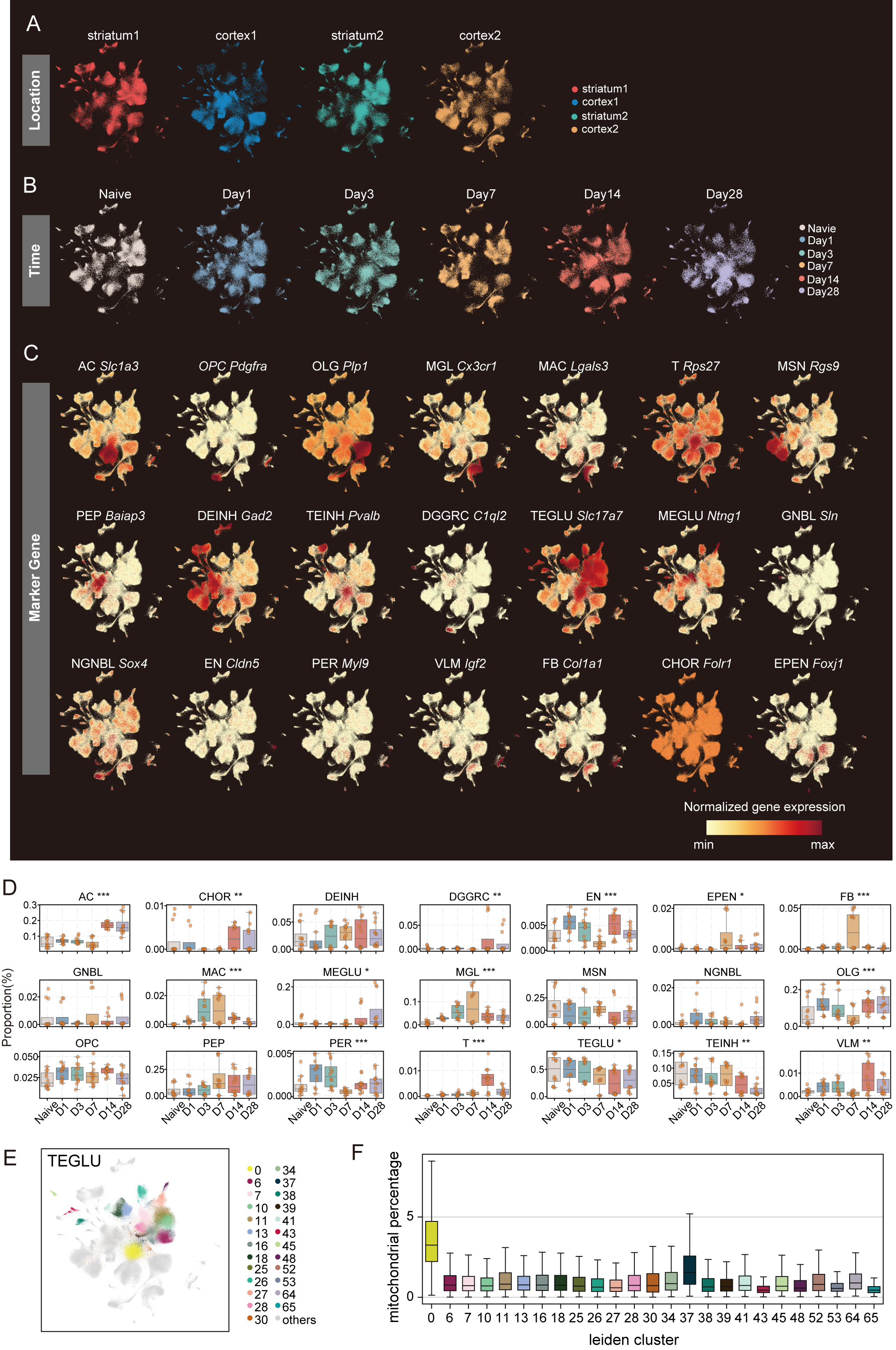

Supplement: Supplementary file 7 — Supporting Information [file CTM2-15-e70486-s010.tif]

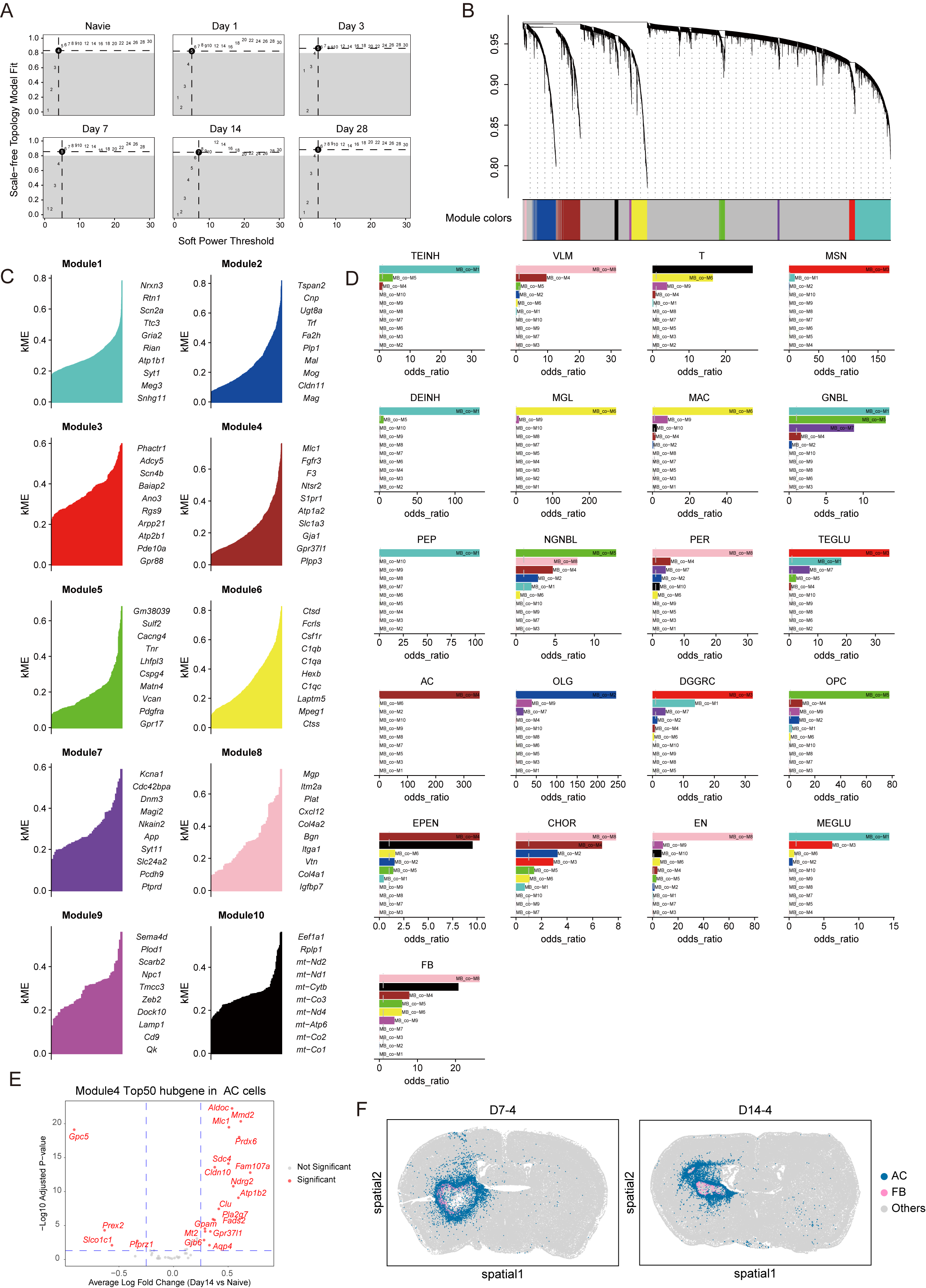

Supplement: Supplementary file 8 — Supporting Information [file CTM2-15-e70486-s003.tif]

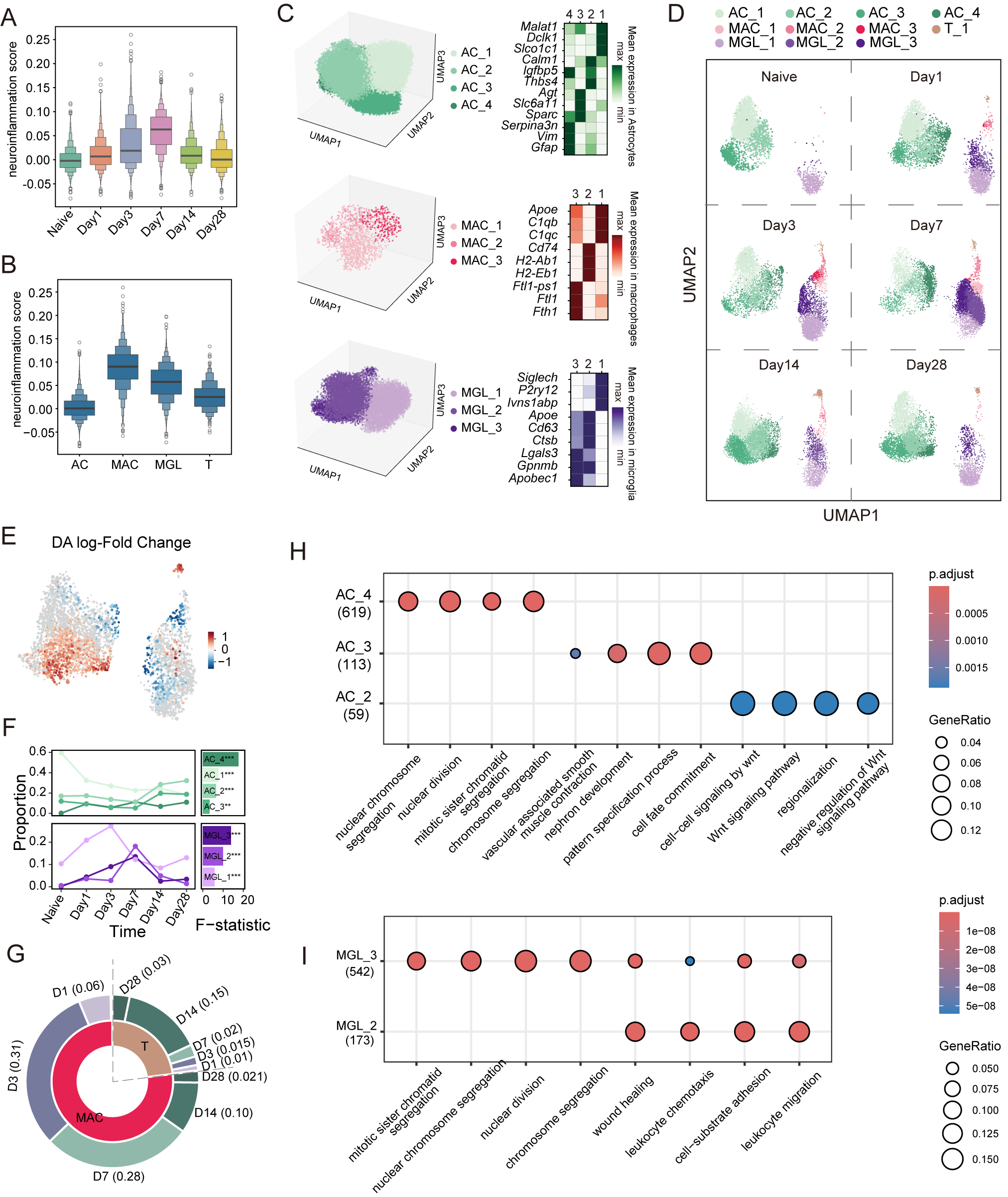

Supplement: Supplementary file 9 — Supporting Information [file CTM2-15-e70486-s007.tif]

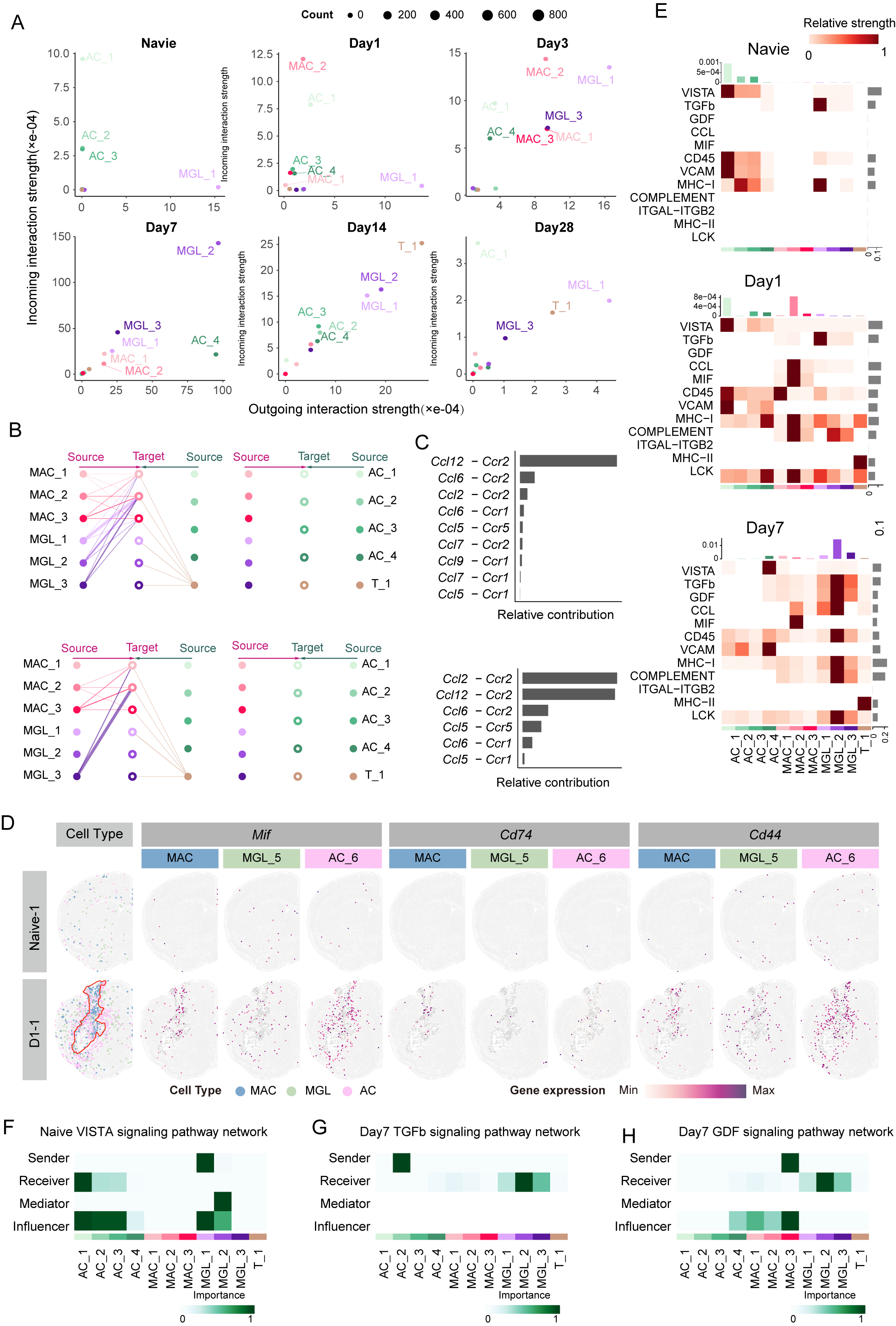

Supplement: Supplementary file 10 — Supporting Information [file CTM2-15-e70486-s011.tif]

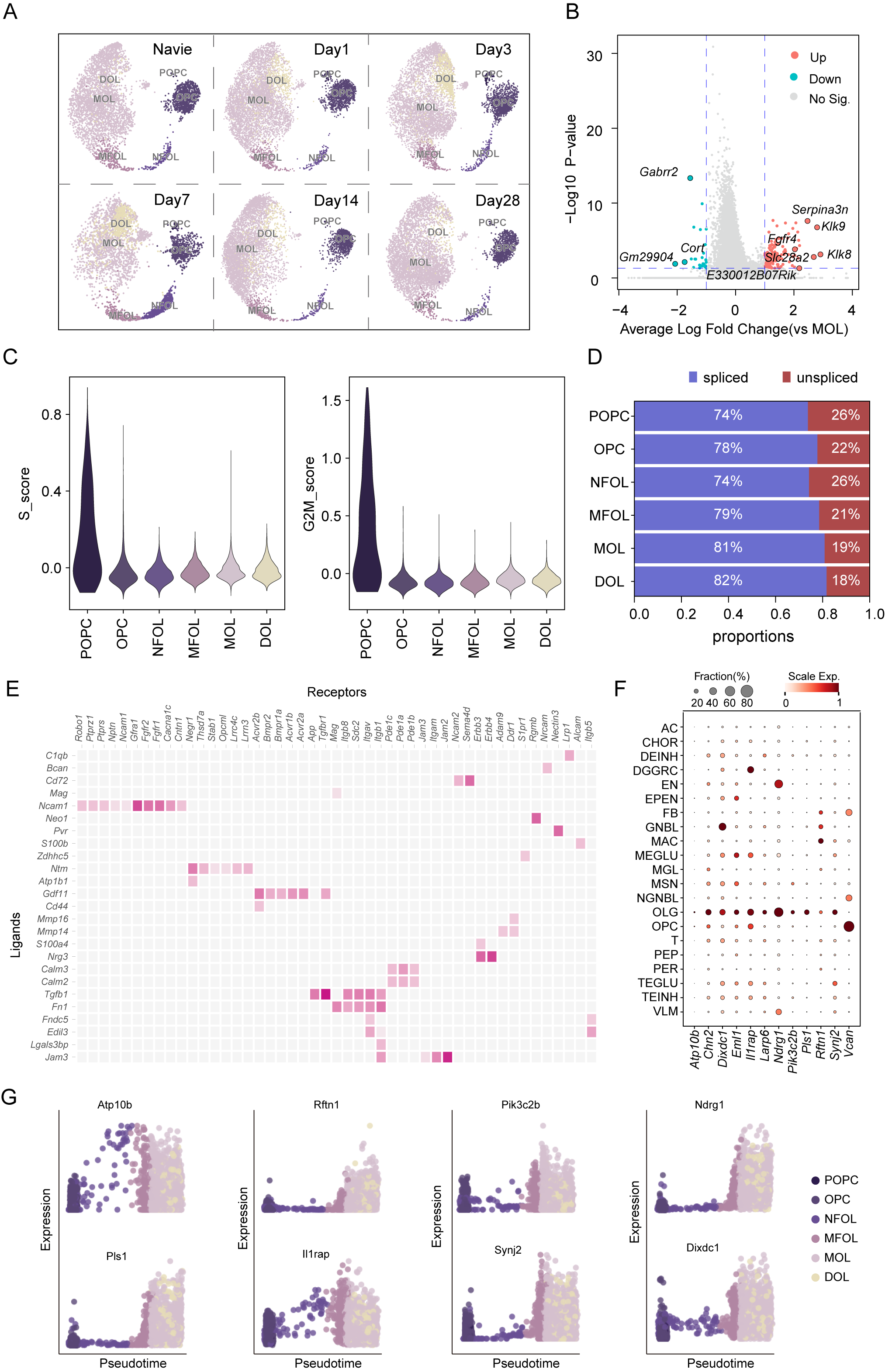

Supplement: Supplementary file 11 — Supporting Information [file CTM2-15-e70486-s001.tif]
